# OP62 Economic Evaluations Submitted For Reimbursement To The Brazilian Unified Health System: A Meta-Epidemiological Study

**DOI:** 10.1017/S0266462324001223

**Published:** 2025-01-07

**Authors:** Maicon Falavigna, Gilson Dorneles, Cintia Araujo, Suena Parahiba, Cinara Stein

## Abstract

**Introduction:**

Allocating financial resources efficiently poses a significant challenge for health systems worldwide. In this context, economic evaluations (EE) are pivotal for the health technology assessment (HTA) process, particularly in standardizing approaches to enhance decision-making accuracy. This study focuses on delineating methodological nuances of EE submissions within Brazil’s Unified Health System (SUS).

**Methods:**

This meta-epidemiological study analyzed dossiers submitted to the National Committee for Health Technology Incorporation (Conitec) for drug reimbursement from January 2022 to May 2023. We selected dossiers that included complete EE studies such as cost–utility or cost-effectiveness analyses. Our evaluation involved extracting data on the characteristics of the studies, reimbursement decisions, elements of the base case scenario, and parameters used in utility and sensitivity analyses. [Protocol: DOI 10.17605/OSF.IO/JVYEC]

**Results:**

We included 56 dossiers, leading to 11 favorable reimbursement decisions. EE study methods were cost-effectiveness (17) and cost–utility analyses (17), with some employing both (16). Markov chain models were used in 27 dossiers, primarily utilizing quality-adjusted life years as the health outcome measure. Additional outcomes included life years gained and frequency of avoided events. While utility assessment was reported in 33 dossiers, only six adjusted for age and seven accounted for disutility from adverse events. Thirty-two conducted deterministic or probabilistic sensitivity analyses, but only four presented parameters like credibility intervals, and 17 presented acceptability curves.

**Conclusions:**

This study highlights the need for more standardized and refined methods in the EE submitted to Conitec. The existing EE methodological guidelines, dating from 2014, are currently under revision. This update is crucial to integrate recent advancements in health technology assessment and to better address contemporary requirements, particularly in light of the newly defined willingness-to-pay threshold.

